# Oxidized macrophage migration inhibitory factor is a potential new tissue marker and drug target in cancer

**DOI:** 10.18632/oncotarget.11970

**Published:** 2016-09-12

**Authors:** Alexander Schinagl, Michael Thiele, Patrice Douillard, Dirk Völkel, Lukas Kenner, Zahra Kazemi, Michael Freissmuth, Friedrich Scheiflinger, Randolf J. Kerschbaumer

**Affiliations:** ^1^ Baxalta Innovations GmbH, Orth/Donau, Austria; ^2^ Department of Pathology, Medical University Vienna, Vienna, Austria; ^3^ Ludwig Boltzmann Institute for Cancer Research, Vienna, Austria; ^4^ Institute of Laboratory Animal Pathology, University of Veterinary Medicine Vienna, Vienna, Austria; ^5^ Institute of Pharmacology, Centre of Physiology and Pharmacology, Medical University, Vienna, Austria

**Keywords:** MIF, cancer, inflammation, antibodies, drug target

## Abstract

Macrophage migration inhibitory factor (MIF) is a pleiotropic cytokine, which was shown to be upregulated in cancers and to exhibit tumor promoting properties. Unlike other cytokines, MIF is ubiquitously present in the circulation and tissue of healthy subjects. We recently described a previously unrecognized, disease-related isoform of MIF, designated oxMIF, which is present in the circulation of patients with different inflammatory diseases. In this article, we report that oxMIF is also linked to different solid tumors as it is specifically expressed in tumor tissue from patients with colorectal, pancreatic, ovarian and lung cancer. Furthermore, oxMIF can be specifically targeted by a subset of phage display-derived fully human, monoclonal anti-MIF antibodies (mAbs) that were shown to neutralize pro-tumorigenic activities of MIF *in vivo*. We further demonstrate that anti-oxMIF mAbs sensitize human cancer cell lines (LNCaP, PC3, A2780 and A2780ADR) to the action of cytotoxic drugs (mitoxantrone, cisplatin and doxorubicin) *in vitro* and in an A2780 xenograft mouse model of ovarian cancer. We conclude that oxMIF is the disease related isoform of MIF in solid tumors and a potential new diagnostic marker and drug target in cancer.

## INTRODUCTION

Macrophage migration inhibitory factor (MIF) is a pro-inflammatory cytokine that promotes tumor growth and metastasis *in vivo* by multiple modes of action [1–11]. MIF was shown to trigger cell proliferation by activation of the central kinases Akt and ERK, thereby promoting sustained activation and survival of immune cells and cancer cell proliferation [12–14]. Genetic loss of MIF has been described to cause p53-dependent growth alterations, increased p53 transcriptional activity, altered RHO-dependent cyclin D1 expression, and resistance to RAS-mediated oncogenic transformation [15–17]. MIF also plays a key role in angiogenesis and neovascularization: it is associated with hypoxic adaptation and stabilization of hypoxia-inducible factor 1-alpha (HIF-1α) [6]. In this context, MIF was shown to contribute to the up-regulation of vascular endothelial growth factor (VEGF), IL-8 and matrix metalloproteinases (MMPs) [7, 18, 19]. Furthermore, MIF promotes a pro-inflammatory tumor microenvironment (TME) by induction of cytokines and other mediators of inflammation, such as TNF-α [20], nitric oxide [21] and prostaglandin E2 [12]. Tumor associated macrophages (TAMs) and myeloid-derived suppressor cells (MDSCs) from MIF-deficient mice exhibit reduced immunosuppressive activities resulting in improved immune responses against melanoma [22]. Chemokine functions of MIF are expected to play an important role in altering the TME as they contribute to the infiltration of leukocytes into tumors, thereby promoting cancer related inflammation [20, 23].

*In vivo*, genetic knock-out of MIF was shown to blunt tumor outgrowth in animal models of breast cancer [24], skin cancer [25], gastric cancer [26], bladder cancer [27], lung cancer [28] and fibrosarcoma [15]. Blocking of MIF activity either by antibodies or stable RNA interference reduced tumor growth in animal models of colorectal cancer [29], prostate cancer [30], ovarian cancer [20], neuroblastoma [31], pancreatic cancer [10], breast cancer [9], melanoma [22, 32] and lung cancer [28]. The involvement of MIF in human tumor development has been substantiated by reports that describe higher MIF levels in the circulation of cancer patients. Increased circulating MIF levels are correlated with high tumor burden and metastasis in *e.g.* prostate cancer, lung cancer, colon cancer and ovarian cancer [7, 33–35]. MIF was further shown to be upregulated in the tissue of different tumor types, i.e. pancreatic, breast, prostate, colon, brain, skin, and lung tumors [1, 3, 4, 7, 36–38]. However, MIF cannot be considered a tumor specific marker as it is constitutively expressed and secreted by numerous cell types and significant levels of MIF can be found in the tissue and circulation of healthy subjects [39]. At the first glance, this fact makes MIF a challenging target for specific therapeutic intervention.

We recently reported that MIF occurs in two immunologically distinct redox-dependent isoforms, termed oxidized MIF (oxMIF) and reduced MIF (redMIF) [40]. RedMIF was found to be the abundantly expressed isoform of MIF that can be detected even in healthy subjects. In contrast, oxMIF represents the disease-related isoform which was detected predominantly in the circulation and on the surface of cells isolated from patients with inflammatory diseases. The fully human monoclonal anti-oxMIF antibodies BaxB01, BaxG03 and BaxM159 were shown to strictly differentiate between redMIF and oxMIF and to exert *in vivo* protective effects in animal models of inflammation [40, 41]. We therefore sought to investigate the expression of oxMIF in the circulation and in cancer tissue of patients with different types of solid tumors, and to elucidate anti-proliferative effects of oxMIF specific antibodies in combination with cytotoxic drugs.

## RESULTS

### OxMIF can be detected in plasma of patients with solid tumors

**Figure 1:**
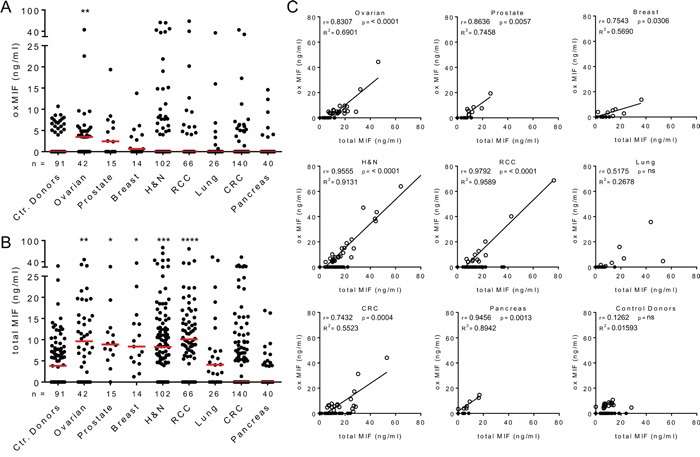
Presence of oxMIF in the circulation of cancer patients and healthy controls **A.** Plasma levels of oxMIF in samples from control donors and patients with solid tumors. **B.** Plasma levels of total MIF in the same samples. Individual values and medians (red lines) are shown. We used the Kruskal-Wallis test followed by Dunn's multiple comparison test for statistical analyses. *p<0.05; **p<0.01***; p<0.001; ****p<0.0001. **C.** OxMIF levels plotted against total MIF levels for each individual plasma sample. OxMIF positive plasma samples (open circles) and oxMIF negative samples (closed circles) are depicted. We used Pearson correlation analysis and linear regression to correlate MIF and oxMIF levels in each cancer type, excluding oxMIF negative samples (oxMIF = 0 ng/ml).

Previous studies described the elevation of MIF in the circulation of cancer patients [7, 33–35]. However, these studies did not discriminate between redMIF and oxMIF. We utilized two previously established ELISA methods [40] to quantify oxMIF and total MIF, which reflects the sum of oxMIF and redMIF, in plasma samples of cancer patients and healthy controls. In the control donor group we detected small amounts of oxMIF (up to 10.7 ng/ml) in 20 out of 91 plasma samples (22% oxMIF positive; median: 0.0 ng/ml) (Figure 1A). OxMIF levels were significantly elevated in plasma samples from patients with ovarian cancer (23/42, 55% oxMIF positive; median: 3.5 ng/ml) compared to plasma samples from healthy controls. In plasma samples obtained from patients with prostate cancer (8/14, 57% oxMIF positive; median: 2.4 ng/ml), breast cancer (8/15, 53% oxMIF positive; median: 0.6 ng/ml), head and neck cancer (27/102, 26% oxMIF positive; median: 0.0 ng/ml), renal cell carcinoma (13/66, 20% oxMIF positive; median: 0.0 ng/ml), lung cancer (7/26, 27% oxMIF positive; median: 0.0 ng/ml), colorectal carcinoma (18/140, 13% oxMIF positive; median: 0.0 ng/ml) and pancreatic cancer (7/40, 18% oxMIF positive; median: 0.0 ng/ml), levels of oxMIF were not significantly different from the control donor group (Figure 1A). Nevertheless, it is noteworthy that oxMIF levels showed a considerable patient to patient variation, and in each cohort, oxMIF levels > 15 ng/ml could be found in some individual plasma samples.

As expected, a baseline level of total MIF was detected in the same plasma samples from control donors (median 3.8 ng/ml), which is consistent with the MIF levels published in the literature [43, 44]. Significantly increased levels of MIF were detected in plasma from patients with ovarian cancer (median 9.6 ng/ml), prostate cancer (median 8.9 ng/ml), breast cancer (median 8.4 ng/ml), head and neck cancer (median 8.3 ng/ml) and renal cell carcinoma (median 10.1 ng/ml) (Figure 1B). Total MIF levels of patients with lung cancer, colorectal carcinoma and pancreatic cancer did not differ significantly from those of healthy controls. Correlation plots demonstrated that plasma levels of oxMIF and total MIF correlated in oxMIF positive patients, whereas no correlation was seen in healthy controls (Figure 1C). When oxMIF was detectable, this isoform comprised approximately 55-95% of MIF in the circulation of patients, which is comparable to the ratio found in patients with inflammatory diseases [40].

Taken together, our data confirm that (total) MIF levels were significantly upregulated in the plasma of patients with solid tumors, such as ovarian cancer [35] or prostate cancer [33]. Circulating oxMIF was significantly elevated in ovarian cancer patients, but not in the other cancer indications tested. Most of the plasma samples from patients with solid tumors did not show detectable amounts of oxMIF. However, oxMIF positive patient subpopulations were identified in all solid tumor indications analyzed, with levels up to 80 ng/ml.

### OxMIF occurs specifically in malignant tissue and can be detected in primary tumors and in metastases of different cancers

**Figure 2:**
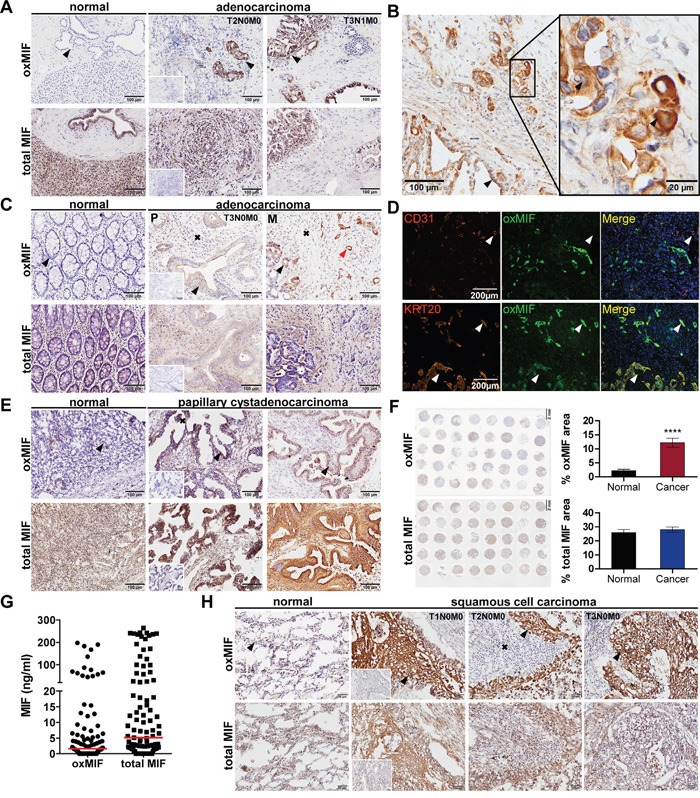
OxMIF occurs specifically in malignant tissue and can be detected in primary tumors and in metastases of different solid tumors **A.** OxMIF and total MIF staining of pancreas tissue with normal morphology and pancreatic adenocarcinoma tissue (tumors were staged according to the tumor node metastasis (TNM) system). **B.** Ten and 40 fold (inset) original magnification of pancreatic adenocarcinoma stained for oxMIF, arrows indicate location of staining (invasion front, membrane, cytoplasm and nucleus, respectively). **C.** OxMIF and total MIF staining of colon tissue with normal morphology and colorectal adenocarcinoma tissue, red arrows indicate vessel like structures described in D. **D.** Top panels show immunofluorescence staining of the blood vessel marker CD31 (red) and oxMIF (green). The overlay reveals no co-localization (white arrow) of these markers in CRC metastasis obtained from the liver. Bottom panels show immunofluorescence staining of the colon epithelial cell marker cytokeratin 20 (KRT20, red) and oxMIF (green). The merge with DAPI nuclear counterstain reveals co-localization (white arrow) of these markers. Scale bar 200 μm. **E.** OxMIF and total MIF staining of ovarian tissue with normal morphology and ovarian papillary cystadenocarcinoma tissue. **F.** The left panels show a low magnification image of a tissue micro array consisting of 37 sections of ovarian cancer tissue and 3 adjacent normal tissues, stained for oxMIF and total MIF. Scale bar 2 mm. This microarray has been analyzed by digital images analysis using Definiens Tissue Studio^®^, and the mean stained tissue area ± SEM are depicted on the right panel. ****p<0.0001, unpaired two-tailed student's t-test. **G.** Levels of oxMIF and total MIF in ascitic fluid from patients with ovarian cancer. Data are presented as dot-plot of individual samples with median (red lines). **H.** OxMIF and total MIF staining of lung tissue with normal morphology and lung cancer tissue as indicated. DAB staining and hematoxylin counterstaining. Scale bars 100 μm (if not otherwise indicated). Black arrows and black crosses indicate epithelial cells (in normal tissue) or tumor epithelial cells and tumor stroma respectively, small insets show control staining with matched non-immune isotype IgG.

MIF has been described to be upregulated in tumor tissue [1, 3, 4, 7, 31, 34, 36–38]. Again, these studies did not differentiate between oxMIF and redMIF. Hence, we analyzed tumor tissue from pancreatic, colorectal, ovarian and lung cancer patients for the presence of oxMIF. However, denaturation of MIF - *i.e.* by fixation - leads to irreversible changes in the MIF structure which results in binding of oxMIF specific antibodies and does not allow a differentiation between redMIF and oxMIF [40]. Therefore, conventional immunohistochemistry (IHC) techniques including tissue fixation steps cannot be applied to detect oxMIF in tissue. To avoid this problem we developed an IHC method, which allows for the specific detection of oxMIF, by using fresh frozen tissue sections and avoiding any fixative prior to incubation with anti-oxMIF antibodies. By using this IHC technique, we analyzed tissue derived from patients with pancreatic ductal adenocarcinoma (PDAC). We observed moderate to strong oxMIF immunostaining in pancreatic intraepithelial neoplasias (PanINs) even at an early tumor stage, *i.e*. stage I-II. Staining was more pronounced in later stage tumors, *i.e*. stage III, with a prominent staining of the invasion front (Figure 2A, middle and right upper images). Adjacent normal pancreatic tissue did not show immunoreactivity for oxMIF (Figure 2A, upper left image). Unlike oxMIF, total MIF was widely expressed in both, PDAC and adjacent normal pancreas tissue (Figure 2A, lower panel), as expected from the literature [38]. At higher magnification it is obvious that oxMIF was predominantly located at the membrane and the cytoplasm of tumor cells, but was also detected in some nuclei (Figure 2B). Only weak oxMIF staining was detected in the tumor stroma and some infiltrating immune cells like macrophages (Figure 2B).

We next assessed oxMIF expression in tumor tissue from colorectal cancer (CRC) patients, including primary tumors and liver metastases. We observed moderate cytoplasmic and membranous staining for oxMIF in tumor cells and stroma including some nuclei (Figure 2C, middle and right upper images). A pronounced oxMIF staining was also detected in vessel like structures. We therefore probed consecutive slides of CRC liver metastases for cytokeratin 20 (marker for colon epithelial cells), CD31 (endothelial cell marker) and oxMIF. Co-localization of cytokeratin 20 and oxMIF was detected by immunofluorescence microscopy indicating that these oxMIF positive vessel like structures originated from colorectal cancer cells (Figure 2D, lower panel) and were not part of the tumor stroma originating from adjacent liver tissue (Figure 2D, upper panel). Adjacent normal colon mucosa and liver tissue did not show immunoreactivity for oxMIF (Figure 2C, upper left image). In contrast, total MIF was widely expressed in both, CRC (primary and metastatic) derived tissue and adjacent colon mucosa (Figure 2C, lower panel).

We further investigated oxMIF expression in ovarian cancer tissue: weak to strong cytoplasmic and membranous oxMIF staining, depending on the tumor type was observed in apical papillary tumor cells and in cells within the papillary projections as well as in the tumor stroma (Figure 2E, upper panel). The strongest staining was evident in adenocarcinoma, serous adenocarcinoma and mucinous cystadenocarcinoma (Figure 2E and data not shown). OxMIF was not detected in normal ovarian tissue (Figure 2E, upper left image). In addition, we assessed the expression of oxMIF in a customized ovarian cancer tissue micro array (TMA) including 37 ovarian cancer cores and 3 normal ovarian tissue cores. By applying digital image analysis (DIA) single tumor cores were analyzed and the stained tissue area was calculated after accounting for background staining. The result of this DIA demonstrated that oxMIF was significantly overexpressed in ovarian cancer compared to normal ovarian tissue (Figure 2F, upper panel; Supplementary Figure S1). Total MIF showed a moderate to strong uniform staining in each of the tumor cores (tumor cells and stroma) as well as in normal ovarian tissue (Figure 2E, lower panel). DIA revealed no difference in total MIF expression between normal and cancerous tissue (Figure 2F, lower panel; Supplementary Figure S1). We furthermore analyzed ascites fluid from ovarian cancer patients by ELISA to determine the amount of oxMIF and total MIF. OxMIF levels varied from 0 ng/ml to amounts as high as 200 ng/ml (67/99, 68% oxMIF positive; median: 1.6 ng/ml). Total MIF levels were slightly higher and ranged from 0 ng/ml to 260 ng/ml (median: 5.1 ng/ml) (Figure 2G).

Finally, we examined several sections of fresh frozen tumor blocks from non-small cell lung cancer (NSCLC) patients and adjacent non-neoplastic lung epithelium for the presence of oxMIF. We detected oxMIF (weak to strong cytoplasmic staining) in most of the lung cancer samples. Staining intensity varied between the different types of NSCLC, with most prominent staining in adenocarcinomas and squamous cell carcinomas (Figure 2H, upper panel). In some samples, patches of cytoplasmic oxMIF staining were visible in apical tumor cells and oxMIF immunostaining was also apparent in the stroma (Figure 2H, upper panel). For total MIF, we again detected a moderate to strong uniform staining in tumor cells and tumor stroma of most tumor cores as well as in normal lung tissue (Figure 2H, lower panel). No immunoreactivity was seen in tissue sections that were incubated with human or rabbit matched isotype IgG control antibodies (Figure 2A, 2C, 2E, 2H – insets in middle/second panels).

The IHC analyses demonstrate that oxMIF can be specifically detected in different cancer tissues, whilst it is not detectable in adjacent non-tumorous tissue. Therefore, this study provides new evidence that oxMIF is a promising tissue marker for diagnostic purposes in solid cancers and can be detected prominently at primary tumor sites and corresponding metastases.

### Anti-oxMIF mAbs sensitize cancer cells to the action of cytotoxic drugs *in vitro* and *in vivo*

We have previously described that the fully human antibodies BaxB01, BaxG03 and BaxM159 are specific for oxMIF and do not bind to redMIF [40]. We furthermore described that these three antibodies were able to reduce cell growth and viability of prostate cancer cell lines *in vitro* and *in vivo* in a monotherapeutic setting [42], whereas a fully human antibody that does not discriminate between oxMIF and redMIF failed to exert significant beneficial anti-tumor effects *in vivo* (data not shown). Mechanistically, these anti-oxMIF antibodies inhibited proliferation and survival signaling pathways and reduced the level of active ERK1/2 and active Akt, and led to an accumulation of active caspase 3 [42], which is in line with reports on the effects of neutralizing biologic activity of MIF [12, 14].

**Figure 3:**
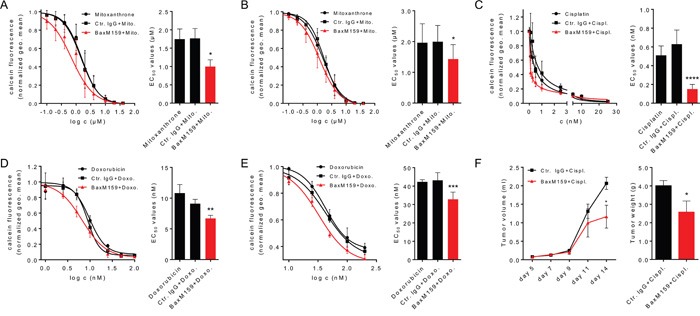
Anti-oxMIF mAbs sensitize cancer cells to cytotoxic drugs *in vitro* and *in vivo* Prostate cancer cell lines LNCaP **A.** or PC3 **B.** were incubated with various concentrations of mitoxantrone (0.01-40 μM) either in the presence of 100 nM BaxM159, or matched human isotype control antibody (Ctr. IgG) or without antibody. The ovarian cancer cell line A2780 was incubated with various concentrations of cisplatin (0.1-25 nM) **C.** or doxorubicin (3.13-200 nM) **D.** either in the presence of 50 nM BaxM159, or matched human isotype control antibody (Ctr. IgG) or without antibody. The adriamycin-resistant ovarian cancer cell line A2780ADR **E.** was incubated with various concentrations of doxorubicin (3.13-200 nM) either in the presence of 50 nM BaxM159, or matched human isotype control antibody (Ctr. IgG) or without antibody. After 48 h cells were labeled with calcein-AM and live cells were counted by flow cytometry. EC50 values for cytotoxic drugs were calculated by fitting the data points to a four-parameter variable slope equation (Hill-equation). Curve fits (left panels) and EC50 values (right panels) are represented as means ± SEM from at least 4 independent experiments. *p<0.05; **p<0.01; ***p<0.001; ****p<0.0001. We used one-way ANOVA followed by Dunnett's multiple comparison test. **F.** MF-1 nude mice (n= 10/group) were inoculated with 1 × 10^6^ A2780 cells suspended in matrigel. Mice were treated with cisplatin (2.5 mg/kg) and BaxM159 (15 mg/kg) or human Ctr. IgG (15 mg/kg). Tumor volumes were measured at indicated time points. On day 14, mice were sacrificed, the tumors excised and weighed. Data are shown as means ± SEM. *p<0.05. We used student's unpaired t-test.

Due to narrow therapeutic indices, toxicities and development of tumor resistance to chemotherapeutic drugs, current treatment strategies include combinations of targeted therapy with one or more chemotherapeutic agents. Examples include trastuzumab (anti-HER2/neu) in combination with paclitaxel in breast cancer [45], rituximab (anti-CD20) in combination with cyclophosphamide/doxorubicin/vincristine/dexamethasone in non-Hodgkin's lymphoma [46] or cetuximab (anti-EGFR) in combination with irinotecan in colon cancer [47]. We assessed whether the combination of anti-oxMIF antibody BaxM159 and chemotherapeutics have synergistic effects on tumor cell growth inhibition compared to single agents *in vitro* and *in vivo*. As shown in Figure 3, sensitization of the prostate cancer cell lines LNCaP (androgen-receptor positive) or PC3 (androgen-receptor negative) with BaxM159 significantly reduced the half-maximal effective concentration (EC_50_) of mitoxantrone by 40% and 30%, respectively (Figure 3A and Figure 3B). In addition, we found that BaxM159 also sensitized the ovarian cancer cell line A2780 to cisplatin and doxorubicin (= adriamycin). *In vitro*, BaxM159 reduced the EC_50_ of cisplatin and doxorubicin by about 70% and 30%, respectively (Figure 3C and Figure 3D). Of note, BaxM159 was also able to significantly sensitize the adriamycin-resistant ovarian cancer cell line A2780ADR to the action of doxorubicin by reducing the EC_50_ by 25% (Figure 3E). We next sought to translate this *in vitro* synergistic effect of combinatory treatment into an *in vivo* setting. Mice bearing A2780 ovarian xenografts were treated with 2.5 mg/kg cisplatin once a week, either in combination with 15 mg/kg BaxM159 or with 15 mg/kg isotype control IgG every other day. Combination of cisplatin with BaxM159 resulted in significantly reduced final tumor weights (~ 45%) when compared to cisplatin and irrelevant control antibody treated mice (Figure 3F). Our results demonstrate the capability of anti-oxMIF mAbs to sensitize cancer cells to cytotoxic agents which results in an improved anti-tumorigenic effect.

## DISCUSSION

We recently described the identification of oxMIF, a disease-related isoform of MIF that is predominantly expressed in patients with inflammatory diseases, whereas redMIF represents the ubiquitous isoform of MIF that is abundantly expressed even in healthy subjects. In inflammatory diseases, oxMIF was detected in the circulation of patients, therefore representing a systemic marker of inflammation [40]. In contrast, in cancer indications plasma levels of oxMIF were significantly elevated in samples from ovarian cancer patients, but not in patients with other solid tumor types investigated.

We furthermore described that oxMIF expression is linked to sites of inflammation [40]. This linkage of oxMIF expression to diseased tissue seems to be particularly pronounced in cancer indications, as oxMIF is specifically expressed in the tissue of all human cancer types investigated. Remarkably, oxMIF was detected in tissue of early stage adenocarcinomas of pancreatic and colon cancer, as well as in early stage lung and ovarian tumors. In many ovarian cancer patients oxMIF furthermore accumulated in abdominal ascites, supporting the assumptions that oxMIF expression is linked to the tumor tissue and that oxMIF is released during disease progression. Immunofluorescence analyses of liver metastases proved that oxMIF expression originated from colon epithelial tumor cells. In view of recent findings where MIF was shown to be a key mediator of PDAC metastasis [10], oxMIF is likely to play a role in liver niche formation and CRC metastasis.

A targeted MIF-directed therapy seems to be challenging due to high MIF plasma levels and its ubiquitous expression in tissue and cells with normal morphology. The specific expression of oxMIF in cancerous tissue allows for targeted inhibition of disease-related functions of MIF in cancer. Anti-oxMIF antibodies BaxB01, BaxG03 and BaxM159 were shown to inhibit growth of prostate cancer cells *in vitro* and *in vivo* [42]. Here we demonstrated that anti-oxMIF antibody BaxM159 was capable of sensitizing prostate and ovarian cancer cells to the action of cytotoxic drugs *in vitro*. Combining cisplatin with BaxM159 also increased the cytotoxicity of cisplatin *in vivo*, which resulted in reduced A2780 tumor growth rates in mice. A fully human anti-MIF antibody which does not distinguish between redMIF and oxMIF failed to exert a significant beneficial effect in a mouse xenograft prostate cancer model (results not shown). This is in line with the previously reported observation that recombinant human antibodies that do not discriminate between the two isoforms failed to show beneficial therapeutic effects in animal models of inflammation [40].

Reduction of the level of activated kinases Akt and ERK1/2 and activation of caspases were previously described as underlying mechanisms for the anti-tumor effects of anti-oxMIF antibodies [42]. As oxMIF could be detected on the cellular surface and in the cytoplasm of tumor cells, it will be of interest to discover whether anti-oxMIF antibodies interfere with extracellular and/or intracellular functions of MIF which convert into an inhibition of Akt and ERK1/2 signaling and in an activation of caspases. An interference of anti-oxMIF antibodies with intrinsic MIF activities is conceivable as an increased caspase activity could be mediated not only via Akt but also via the MIF-p53 axis [15]. Anti-oxMIF antibodies induce neither complement-dependent cell lysis nor antibody-dependent cellular cytotoxicity. Therefore, the beneficial effect of down regulation of these kinases might have been enhanced by the direct cytotoxic effects of cisplatin, doxorubicin and mitoxantrone. It will also be important to identify immunomodulatory activities of anti-oxMIF antibodies in the tumor microenvironment as processes of stromal remodeling could be crucial for an increased cytotoxicity of chemotherapeutics when administered in combination with anti-oxMIF antibodies. An interference of anti-oxMIF antibodies with pro-angiogenic properties of MIF might also be a part of the mechanism of action and should therefore be addressed in future investigations. Furthermore, the exact structural basis for the rearrangement of redMIF to the diseased related isoform oxMIF as well as MIF binding proteins and receptors that are involved in regulating this transition are currently unknown and require further investigations.

Taken together, we demonstrated that oxMIF is a new therapeutic target in solid tumors and that anti-oxMIF antibodies are a new class of potent inhibitors of MIF-related functions in tumorigenesis with a promising use in combination therapy. We also highlight oxMIF as a compelling new tissue marker which might be important for diagnostic and prognostic purposes, especially in combination with treatment strategies.

## MATERIALS AND METHODS

### Reagents

BaxB01, BaxM159, and an isotype-matched human control antibody were produced as described [41]. Monoclonal rabbit anti-MIF antibodies were generated by immunization of rabbits with recombinant MIF, spleens were resected and B-cells were used for RabMab-Hybridomatechnology (Epitomics, Abcam). High affinity hybridoma clones were selected and RabMab IgGs were purified from cell culture supernatant over protein A columns. Recombinant MIF was expressed in *E. coli* and purified as described [41]. Mitoxantrone, cisplatin and doxorubicin were obtained as commercial formulations approved for human use (Ebexantron^®^, Platinol^®^, Adriblastin^®^).

### Human sample collection

Plasma samples from healthy subjects (control donors) were collected from volunteers in a normal state of health, with no apparent signs of disease. Plasma samples from control donors and cancer patients were purchased either from Tissue Solutions Ltd., Asterand Bioscience Inc., Cureline Inc. or Biochemed Services Inc. Frozen tissue samples and frozen tissue micro arrays were acquired from Asterand Bioscience Inc. and Biochain Inc., respectively. All patients and control donors signed an informed consent for sample collection.

### A2780 ovarian cancer xenograft mouse model

Human ovarian cancer cells A2780 were harvested from exponentially growing cultures and mixed with growth factor-depleted matrigel (BD); 1 × 10^6^ cells in 0.25 ml matrigel were then inoculated subcutaneously into the right flank of female MF1 nude mice. One day after inoculation, treatment (n=10 in each animal group) was started by i.p. administration of the indicated doses of BaxM159 and control antibody. Repetitive administration of antibodies was done every other day. Cisplatin was administered i.p. once per week at a dose of 2.5 mg/kg. Treatment with cisplatin was started one day after the first antibody administration. The sizes of tumor xenografts were measured when their growth became evident (typically on day 7) and volumes were calculated using the formula V=0.5*a*b^2^ (where “a” is the longest diameter and “b” is the shortest diameter). Animal experiments were carried out in accordance with the guidelines set forth by the Medical University of Vienna (MUW) (Good Scientific Practice Manual) and were approved by the Animal Welfare Committee of the Medical University of Vienna (Tierversuchskommission) and the Austrian Ministry of Science and Research.

### Cellular assays

Cultures of human prostate cancer cell lines PC3 and LNCaP as well as of the human ovarian cancer cell lines A2780 and A2780ADR were maintained in RPMI 1640 medium supplemented with both 10% fetal calf serum and 2 mM glutamine. Cells were grown at 37°C in a humidified incubator with 5% CO_2_. The indicated concentrations of mitoxantrone, cisplatin or doxorubicin and/or antibodies were added and the cells were incubated for 48 h. Thereafter, the cells were detached with Accutase^®^, pelleted by centrifugation and resuspended in ice cold phosphate-buffered saline (PBS) containing 50 nM calcein-AM. Flow cytometry (10,000 events recorded per measurement; n=3 per determination) was performed on a Becton Dickinson FACScan^®^ with forward scatter, sideward scatter and calcein fluorescence recorded in individual channels. Analysis was done by WinMDI 2.9^®^ software. The geometric mean of the calcein fluorescence was extracted and used as the parameter for further statistical analysis.

### ELISAs

Quantitative determination of oxMIF and total MIF was done as described previously [40].

### Immunohistochemistry

To detect oxMIF, fresh frozen tissue slides were air dried, blocked (BB: 20% goat serum/2% BSA/0.2% fish gelatin) and incubated with biotinylated primary antibody (biotinylated BaxB01 or biotinylated non-immune human IgG, both 5μg/ml) diluted in TBS containing 2% (w/v) BSA and 0.2% (w/v) fish gelatin (PADB). The slides were fixed in 10% (w/v) PBS buffered formalin solution (VWR) and permeabilized in 0.1% TritonX-100 in TBS for prior addition of the enzyme block solution (Dual Endogenous Enzyme Block, Dako). Sections were incubated with RTU Vectastain ABC reagent (Vector Laboratories) and developed with ImmPact DAB substrate (Vector Laboratories). Alternatively, non-labeled BaxB01 (1 μg/ml, prior-fixation) and monoclonal rabbit-anti-BaxB01 antibodies (0.5 μg/ml) in combination with goat anti-rabbit-HRP (Thermo Scientific, 31460, 1:500) were used to detect oxMIF. Following chromogenesis, the sections were washed in tap water, counterstained with hematoxylin (Vector Laboratories), dehydrated in ethanol, cleared in xylene and coverslips were mounted with Vecta^®^Mount permanent mounting medium (Vector Laboratories).

To detect total MIF, fresh frozen tissue slides were fixed in 10% PBS buffered formalin (VWR) and permeabilized by incubating the sections with 0.1% TritonX-100 in TBS. Endogenous peroxidases were blocked by incubating the tissue sections with dual endogenous peroxidase block (Dako). Unspecific binding was blocked with BB. The sections were incubated with monoclonal rabbit anti-MIF antibodies (1 μg/ml) or commercial polyclonal rabbit anti-MIF antibodies (FL115 Santa Cruz; 1:200) and rabbit IgG isotype control diluted in PADB. The staining was done using goat anti-rabbit-HRP (Thermo Scientific) diluted in PADB or ImmPRESS HRP Anti-Rabbit Ig (Vector Laboratories). The Liquid DAB+ Substrate Chromogen System (Dako) was used for chromogenic reaction. Counterstaining and mounting was done as described above. Full slide scans were acquired using an Olympus VS120 slide scanning microscope at 20-fold magnification (UPLANSAPO 20x, NA 0.75; PIKE F505/C Camera, Allied Vision Technologies). Pictures are presented at 10-fold original magnification. Analyses and evaluation and quantification of stainings were performed by a board certified pathologist (L. K.). Digital image analysis (DIA) was performed with Definiens Tissue Studio^®^ v3.6 program.

### Immunofluorescence

Fresh frozen tissue slides were air dried, blocked with BB and incubated with BaxB01 (4 μg/ml, in PDAB) for oxMIF detection. The specimen was fixed in 10% PBS buffered formalin (VWR). Furthermore, the sections were incubated with monoclonal mouse anti-Cytokeratin 20 (Dako M7019, 1:50) or anti-CD31 antibodies (e-bioscience 13-0319, 1:200), and monoclonal rabbit anti-BaxB01 antibodies (0.5 μg/ml) diluted in PADB+0.25% TritonX-100. Rabbit and mouse antibodies were detected by Alexa Fluor^®^ conjugated secondary antibodies (Life Technologies A11034 and A21424, 1:2000) diluted in PADB+0.25% TritonX-100. The slides were rinsed in PBS and coverslips were mounted with ProLong Gold Antifade reagent with DAPI (Life Technologies). Pictures were taken at 20-fold magnification (LUCPlanFLN 20x, NA 0.45) with an Olympus inverted microscope (VS81) using a mercury lamp and the filters DAPI (U-MWU2), FITC (U-MWIBA3) and TRITC (U-MWIGA3), and a XM10 camera (Olympus).

### Statistics

Distributions were evaluated by Kolmogorov-Smirnov test. If normal distribution was confirmed, data were evaluated by one-way ANOVA followed by Dunnett's multiple comparison. Otherwise data were evaluated by Kruskal-Wallis test followed by Dunn's multiple comparison. Two groups were compared by unpaired two-tailed student's t-test (normal distribution) or Mann-Whitney-test. Correlation analysis was done using Pearson correlation analysis and linear regression.

## SUPPLEMENTARY MATERIALS FIGURES


