# Supplementary material for: Oxidized macrophage migration inhibitory factor is a potential new tissue marker and drug target in cancer
Source: Oncotarget. 2016 Sep 12;7(45):73486–96. doi: 10.18632/oncotarget.11970 (PMC5341993; doi:10.18632/oncotarget.11970)
Supplement: Supplementary file 1 [file oncotarget-07-73486-s001.pdf]

Oxidized macrophage migration inhibitory factor is a potential new tissue marker and drug target in cancer

SUPPLEMENTARY FIGURE

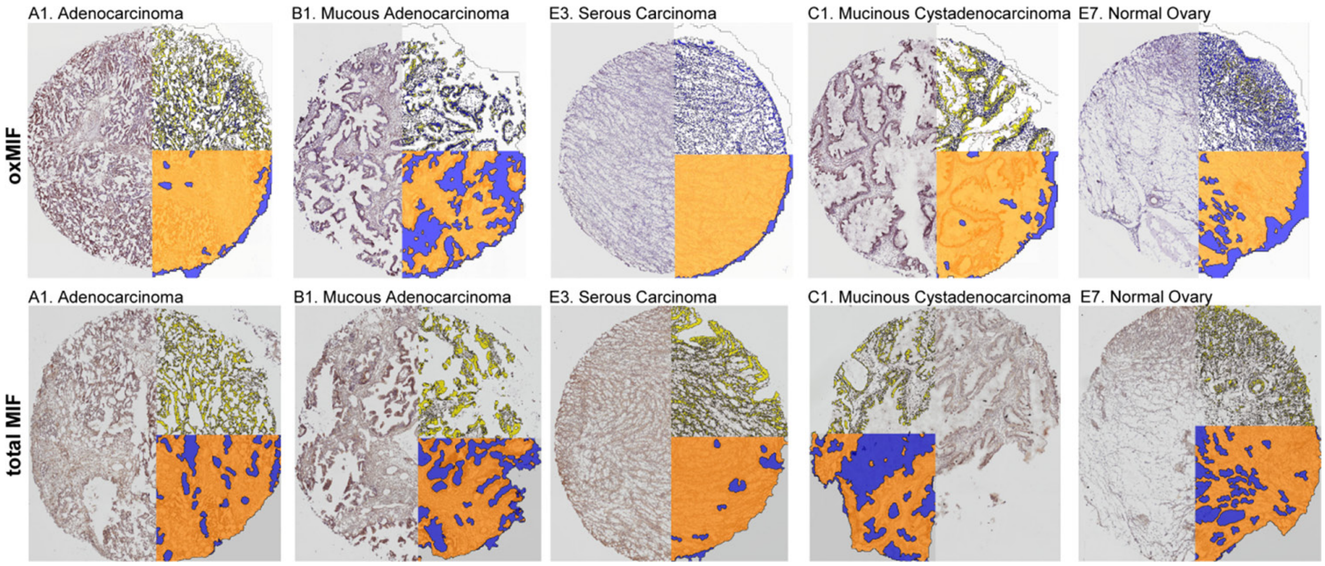

Supplementary Figure S1: High magnification images of selected cores of an ovarian cancer tissue micro array (TMA). A selection of tissue cores depicted in Figure 2F of the manuscript is shown in high magnification. Full slide scans of the TMAs were analyzed by digital image analysis using the Definiens Tissue Studio® v3.6 program. No-tissue areas (blue color) were separated from tumor tissue (orange color) (shown at the bottom right of each core). Nuclei (blue color) and stained areas indicating oxMIF and total MIF (yellow color) were automatically detected by the software (shown on the top right of each core). The bar plots illustrating the mean stained tissue area ± SEM are depicted in Figure 2F of the manuscript.
